# Subjective and Objective Measures of Dryness Symptoms in Primary Sjögren's Syndrome: Capturing the Discrepancy

**DOI:** 10.1002/acr.23165

**Published:** 2017-10-09

**Authors:** Oriana M. Bezzina, Peter Gallagher, Sheryl Mitchell, Simon J. Bowman, Bridget Griffiths, Victoria Hindmarsh, Ben Hargreaves, Elizabeth J. Price, Colin T. Pease, Paul Emery, Peter Lanyon, Michele Bombardieri, Nurhan Sutcliffe, Costantino Pitzalis, John Hunter, Monica Gupta, John McLaren, Anne M. Cooper, Marian Regan, Ian P. Giles, David A. Isenberg, Vadivelu Saravanan, David Coady, Bhaskar Dasgupta, Neil J. McHugh, Steven A. Young‐Min, Robert J. Moots, Nagui Gendi, Mohammed Akil, Kirsten MacKay, W. Fai Ng, Lucy J. Robinson, the UK Primary Sjögren's Syndrome Registry, Elalaine C Bacabac, Robert Moots, Kuntal Chakravarty, Shamin Lamabadusuriya, Constantino Pitzalis, Rashidat Adeniba, John Hamburger, Andrea Richards, Saaeha Rauz, Sue Brailsford, Joanne Logan, Diarmuid Mulherin, Jacqueline Andrews, Alison McManus, Colin Pease, Alison Booth, Theodoros Dimitroulas, Lucy Kadiki, Daljit Kaur, George Kitas, Mark Lloyd, Lisa Moore, Esther Gordon, Cathy Lawson, Lesley Stirton, Gill Ortiz, Elizabeth Price, Gavin Clunie, Ginny Rose, Sue Cuckow, Susan Knight, Deborah Symmons, Beverley Jones, Shereen Al‐Ali, Andrew Carr, Katherine Collins, Ian Corbett, Christine Downie, Suzanne Edgar, Marco Carrozzo, Francisco Figuereido, Heather Foggo, Claire Humphreys, Katherine James, Dennis Lendrem, James Locke, Iain Macleod, Philip Mawson, Philip Stocks, Jessica Tarn, Adrian Jones, Alice Muir, Paula White, Steven Young‐Min, Susan Pugmire, Saravanan Vadivelu, Annie Cooper, Marianne Watkins, Anne Field, Stephen Kaye, Devesh Mewar, Patricia Medcalf, Pamela Tomlinson, Debbie Whiteside, Neil McHugh, John Pauling, Julie James, Nike Olaitan, Jayne McDermott, Olivia Godia, Elizabeth Kidd, Lynne Palmer, Victoria Katsande, Pamela Long, Charles Li, Usha Chandra, Stefano Fedele, Ada Ferenkeh‐Koroma, Ian Giles, David Isenberg, Helena Maconnell, Stephen Porter, Paul Allcoat

**Affiliations:** ^1^ Institute of Neuroscience Newcastle University Newcastle upon Tyne UK; ^2^ Newcastle upon Tyne Hospitals NHS Foundation Trust Newcastle upon Tyne UK; ^3^ University Hospitals Birmingham NHS Foundation Trust Birmingham UK; ^4^ Great Western Hospitals NHS Foundation Trust Swindon Wiltshire UK; ^5^ Leeds Institute of Rheumatic and Musculoskeletal Medicine University of Leeds Chapel Allerton Hospital NIHR Leeds Musculoskeletal Biomedical Research Unit Leeds Teaching Hospitals Trust Leeds UK; ^6^ Nottingham University Hospitals NHS Trust Nottingham UK; ^7^ Barts and the London NHS Trust and Barts and the London School of Medicine and Dentistry London UK; ^8^ Barts Health London UK; ^9^ Gartnavel General Hospital Glasgow Scotland; ^10^ NHS Fife Whyteman's Brae Hospital Kirkcaldy; ^11^ Royal Hampshire County Hospital Winchester and Portsmouth Hospitals NHS Trust Portsmouth UK; ^12^ Royal Derby Hospital Derby UK; ^13^ University College London Hospitals NHS Foundation Trust London UK; ^14^ Queen Elizabeth Hospital Gateshead; ^15^ Sunderland Royal Hospital Sunderland UK; ^16^ Southend University Hospital Southend UK; ^17^ Royal National Hospital for Rheumatic Diseases Bath UK; ^18^ Portsmouth Hospitals NHS Trust Portsmouth UK; ^19^ Aintree University Hospitals Liverpool UK; ^20^ Basildon Hospital Basildon UK; ^21^ Royal Hallamshire Hospital Sheffield UK; ^22^ Torbay Hospital Torquay UK; ^23^ Musculoskeletal Research Group, Institute of Cellular Medicine & Newcastle NIHR Biomedical Research Centre for Ageing and Chronic Diseases Newcastle University, and Newcastle upon Tyne Hospitals NHS Foundation Trust Newcastle upon Tyne UK

## Abstract

**Objective:**

To develop a novel method for capturing the discrepancy between objective tests and subjective dryness symptoms (a sensitivity scale) and to explore predictors of dryness sensitivity.

**Methods:**

Archive data from the UK Primary Sjögren's Syndrome Registry (n = 688) were used. Patients were classified on a scale from −5 (stoical) to +5 (sensitive) depending on the degree of discrepancy between their objective and subjective symptoms classes. Sensitivity scores were correlated with demographic variables, disease‐related factors, and symptoms of pain, fatigue, anxiety, and depression.

**Results:**

Patients were on average relatively stoical for both types of dryness symptoms (mean ± SD ocular dryness −0.42 ± 2.2 and −1.24 ± 1.6 oral dryness). Twenty‐seven percent of patients were classified as sensitive to ocular dryness and 9% to oral dryness. Hierarchical regression analyses identified the strongest predictor of ocular dryness sensitivity to be self‐reported pain and that of oral dryness sensitivity to be self‐reported fatigue.

**Conclusion:**

Ocular and oral dryness sensitivity can be classified on a continuous scale. The 2 symptom types are predicted by different variables. A large number of factors remain to be explored that may impact symptom sensitivity in primary Sjögrenʼs syndrome, and the proposed method could be used to identify relatively sensitive and stoical patients for future studies.

## Introduction

Primary Sjögren's syndrome (SS) is an autoimmune disorder of unknown etiology that is characterized by dry eyes and dry mouth and is associated with extraglandular systemic symptoms such as fatigue, pain (myalgia and polyarthralgia), and autonomic dysfunction 1. It has an estimated prevalence of 0.01–0.09% 2 and is more common in women (9:1 female:male ratio) 3. The condition has a marked negative impact on health‐related quality of life and social functioning 4.

The medications used to improve extraglandular symptoms are less effective in treating sicca symptoms 5. An important factor for knowledge and treatment is the weak association between the results of objective clinical tests of tear or saliva production and the severity of self‐reported dryness symptoms. This is reflected in the current America European Consensus Group (AECG) classification criteria, which dictate that a primary SS diagnosis be made when individuals fulfill 4 or more of the established criteria, which include both subjective and objective items 6. Understanding the discrepancy between objective and subjective findings may be of importance for improving research into the condition.

Several studies have indicated weak correlations between objective and subjective indices of ocular dryness 7, 8, 9, 10, 11, 12, 13, 14, 15. Although the majority of these studies found that greater subjective symptoms are associated with greater objective severity, 2 observed that lower subjective symptoms were associated with greater objective severity 7, 15, which may be related to the reduced sensation resulting from greater damage to the eye 7. In contrast, the relationship between subjective and objective oral dryness measures seems to be stronger 16, 17, 18, 19, 20, 21, although there are some individuals with subjective xerostomia who show no objective salivary gland dysfunction 17.

Discrepancies between objective and subjective symptoms create a number of dilemmas for clinicians. For example, patients may not receive optimal treatment (those with abnormal test results but few subjective symptoms may be undertreated, whereas those with normal test results but high subjective symptoms may receive interventions that are unlikely to help). Furthermore, it becomes difficult to interpret (lack of) response to treatment, which could be particularly important in clinical trials of novel therapeutic agents. It is therefore important to explore this relationship in greater depth. The path from pathologic change in tissues to perceived distressing symptoms is complex and dependent on a number of factors relating to both the severity of the underlying disease and to concomitant psychosocial factors such as low mood and anxiety 22, 23, 24, 25, 26. Developing a method to differentiate patients on the basis of their sensitivity to symptoms could aid research on the factors that contribute to variability in the distress and disability caused by primary SS and ultimately could contribute to the stratification of patients for particular management pathways.

The present study develops a novel method to define the degree of concordance/discrepancy between objective and subjective findings. This will be used to investigate the relationship between subjective symptoms and objective measures of dry eyes and mouth in people with primary SS to identify factors associated with symptom sensitivity.

**Significance & Innovations**

- This study outlines a novel method for defining concordance between objective signs and subjective symptoms that is independent of units of measurement.
- The method can identify both stoical individuals (high objective signs and low subjective symptoms) and sensitive individuals (low objective signs and high subjective symptoms).
- We explored factors predicting symptom sensitivity and found that pain and fatigue symptoms were the biggest predictors of sensitivity to ocular and oral dryness, respectively.
- Much of the variance in symptom sensitivity remains unexplained; this method could be used in future studies to identify sensitive individuals and investigate a larger number of predictive factors (including further biologic and psychologic measures).

## Patients and methods

### Participants

The present study uses archive data from 688 patients in the United Kingdom Primary Sjögrenʼs Syndrome Registry database (www.sjogrensregistry.org), who were recruited from 30 hospital sites from August 2009 to March 2012 (for full details, see Ng et al 27). All patients fulfilled the AECG classification criteria 6. Patients gave written informed consent to participate, and National Health Service ethics approval was granted for this study from the North West–Haydock National Research Ethics Service committee.

### Patient‐ and clinician‐reported measures

Subjective symptoms were assessed using the European League Against Rheumatism (EULAR) Sjögren's Syndrome Patient‐Reported Index (ESSPRI) sicca scores. This is a validated self‐report measure of symptoms of ocular and oral dryness 28, 29. Patients rated their symptoms over the past 2 weeks on a 0–10 scale, where 10 = maximum imaginable dryness. In addition, there were also items that measured subjective fatigue, mental fatigue, and pain. Patients also self‐reported their medication use and comorbidities.

The EuroQol 5‐dimension 3‐level scale 30 was used to measure quality of life. It includes a simple visual analog scale (VAS) and a time tradeoff value, and lower values indicate better quality of life. Depression and anxiety were measured using the Hospital Anxiety and Depression Scale 31. The EULAR Sjögrenʼs Syndrome Disease Activity Index 29, 32 and Disease Damage Index (ESSDDI) 33 were used to assess the extent of systemic disease activity and damage, respectively.

#### Ocular dryness

For the Schirmer I test, a sterile strip of filter paper was inserted inside the patient's lower eyelid for 5 minutes, after which the level of wetting was measured using a standardized ruler. The average result of both eyes was then calculated. Participants were asked not to use eyedrops for 2 hours prior to testing. Lower scores indicate abnormal tear production and a score of ≤5 mm/5 minutes is considered severe by AECG criteria 6.

#### Oral dryness

To test unstimulated salivary flow (USF), the patient was required to spit saliva into a graduated test tube every minute. This was conducted at normal room temperature and humidity, and participants were asked not to eat/drink/smoke for at least 2 hours beforehand. According to AECG criteria, a quantity of ≤1.5 ml collected over 15 minutes indicates impaired saliva secretion 6.

### Defining discordance

**Figure 1:**
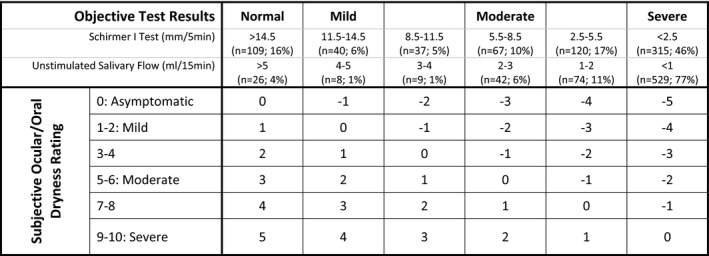
Severity classification groups for objective results and subjective symptoms and the grid used to derive the sensitivity score. For example, an individual with severe objective test results (Schirmer <2.5 or saliva flow <1) but subjectively rating themselves 1 (mild) would have a discrepancy classification of −4, thereby lying at the stoical side of the distribution. An individual subjectively reporting their symptoms 9 (severe) while having a normal objective test results would score +5, at the most sensitive side of the distribution.

The present study used a modified discordance measure that was based on Delbaere et al (2010) 34. Subjective symptom severity and objective test result severity for both ocular and oral dryness were split into classes. Patients’ subjective ocular and oral dryness severities (based on ESSPRI item scores) were grouped into asymptomatic (scoring 0) and symptomatic groups (5 equal classes; see Figure 1).

Objective test result severities were grouped into the same number of classes. As no formal severity “grading” is available for either the Schirmer's test or USF results, reasonable severity “grading” cutoffs, supported by the expert consensus of a consulting rheumatologist (WFN), were established for the purposes of this study, and test results were grouped into equal severity classes, as shown in Figure 1. The severe‐class cutoffs, for both the Schirmer and USF tests, were set as close as possible to the diagnostic cutoffs used in the AECG criteria.

**Figure 2:**
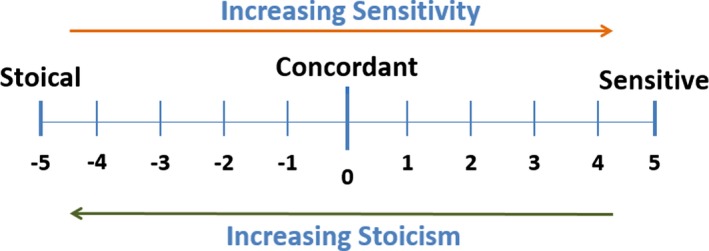
The sensitivity scale used in the study.

The subjective severity classes for ocular and oral dryness were then cross‐tabulated with the corresponding objective severity class in order to identify each patient's degree of sensitivity for ocular and oral dryness. This was done using the sensitivity grid shown in Figure 1. The disparity between subjective symptoms and objective test results was given an arbitrary value and conceptualized on a continuous sensitivity scale (Figure 2). On the scale, a value of 0 signifies full concordance, with negative values indicating increasing stoicism and positive values indicating increasing sensitivity. Patients were grouped into sensitive (positive sensitivity score), accurate (score of 0), and stoical (negative sensitivity score) groups.

### Analysis

Analyses were performed using IBM SPSS software, version 21. Pairwise deletions for missing data were employed. One‐way analysis of variance with post hoc least significant difference tests were used to compare groups, using an alpha level of 0.05. Proportion data between groups were compared using chi‐square tests, with any significant overall difference followed up with pairwise comparisons with a Bonferroni‐adjusted *P* value equaling 0.05 / 3 = 0.017. Bivariate Spearman's correlations were used to explore the relationships between objective and subjective measures and between sensitivity and demographic variables, disease‐ and treatment‐related factors, and self‐ and clinician‐rated symptoms. The strength of correlations was compared using Fisher's r‐to‐z transformation. Linear stepwise hierarchical multiple regression was used to explore predictors of sensitivity. Variables were entered stepwise in the following sequence of blocks: first, demographics and disease factors (age, sex, symptom duration, ESSDDI score, number of comorbidities, number of medications, number of xerogenic medications, and use of a lachrymal/saliva substitute) and, second, other symptoms and quality of life (fatigue, mental fatigue, pain, anxiety, depression, and VAS assessing quality of life). The criteria for entry into the model was a *P* value less than 0.05, and for exit, a *P* value greater than 0.1. Separate regressions were run for ocular and oral sensitivity. Only the results for the variables appearing in the final model are reported.

## Results

Participant characteristics and scores on the measures are shown in Table 1. The majority of the sample was female (n = 651, 95%). Lachrymal and saliva substitutes were used by 544 participants (79.1%) and 305 participants (44.3%), respectively. There were 295 participants (42.9%) who were taking at least 1 xerogenic medication.

**Table 1:** Patient characteristics and variables^a^

	No.	Mean ± SD
Demographics and illness factors		
Age, years	688	58.0 ± 12.5
Disease duration, months	661	80.1 ± 71.5
Symptom duration, months	686	152.4 ± 118.8
Symptom/diagnosis gap, months	659	72.7 ± 98.6
No. comorbidities	688	3.6 ± 2.5
No. medications	688	5.7 ± 4.1
Patient‐rated measures		
Ocular dryness (range 0–10)	681	5.6 ± 2.8
Oral dryness (range 0–10)	681	6.0 ± 2.9
Fatigue (range 0–10)	681	5.5 ± 2.7
Mental fatigue (range 0–10)	680	3.9 ± 2.8
Pain (range 0–10)	680	4.5 ± 3.0
QoL, time tradeoff (range −1.0 to 1.0)	671	0.6 ± 0.3
QoL, VAS (range 0–100)	664	60.3 ± 21.4
HADS anxiety (range 0–21)	666	8.0 ± 4.6
HADS depression (range 0–21)	667	6.0 ± 4.0
Clinician‐rated measures		
ESSDAI (range 0–123)	687	4.8 ± 4.9
ESSDDI (range 0–10)	688	2.5 ± 1.9
Objective tests		
Schirmer's test (mm/5 min)	671	6.2 ± 7.6
Unstimulated salivary flow (ml/15 min)	688	0.9 ± 1.9
Sensitivity		
Ocular sensitivity (range −5 to +5)	681	−0.42 ± 2.2
Oral sensitivity (range −5 to +5)	681	−1.24 ± 1.6

a QoL = quality of life; VAS = visual analog scale; HADS = Hospital Anxiety and Depression Scale; ESSDAI = European League Against Rheumatism (EULAR) Sjögren's Sydrome Disease Activity Index; ESSDDI = EULAR Sjögren's Syndrome Disease Damage Index.

### Subjective versus objective symptoms

A weak but significant correlation (r = −0.13, *P* = 0.001) was found between ocular dryness and Schirmer test results. A moderate correlation (r = −0.31, *P* < 0.001) was found between oral dryness and USF test results. The directions of the relationships indicate that greater symptom severity was associated with lower tear and saliva production. Objective and subjective results were significantly more strongly correlated for oral dryness than for ocular dryness (Z = 3.47, *P* < 0.001).

### Symptom severity and sensitivity

Figure 1 shows the proportion of patients falling into the 6 severity classes on each of the objective measures. Forty‐six percent of patients were in the most severe range for the Schirmer test and 77% for USF, indicating that markedly reduced saliva production was more common than markedly reduced tear production. In what we defined as the normal range for the Schirmer test, there were 16% of patients, compared with only 4% for USF. Cross‐tabulating ocular and oral severity gradings showed that only 8 people (1.2%) were in the normal range on both measures, whereas 268 (39.4%) were in the most severe classification for both. In this sample, a high proportion of patients had severe symptoms on at least 1 objective test measure.

A level of discordance (i.e., a sensitivity score other than 0) was observed in 80.9% and 73.7% of the participants for ocular and oral dryness, respectively. The mean ± SD ocular sensitivity was found to be −0.42 ± 2.2 and −1.24 ± 1.6 for oral sensitivity, indicating that, on average, patients were relatively stoical for both types of dryness symptoms. Fewer patients scored in the sensitive range (≥+1) for oral dryness (n = 59, 8.7%) than for ocular dryness (n = 178, 26.8%). This is partly related to differences in the severity of objective results; the majority of patients had severely reduced saliva production and therefore could not score in the sensitive range. Sensitivity for ocular and oral dryness were positively correlated (r = 0.35, *P* < 0.001), indicating that higher sensitivity for ocular dryness was associated with higher sensitivity for oral dryness.

### Factors influencing ocular and oral dryness sensitivity

Table 2 shows means by group for selected factors that might contribute to symptom sensitivity. The pattern was very similar for both ocular and oral sensitivity. There were no significant differences in total number of comorbidities, but oral‐stoical patients were taking significantly fewer medications (of any type) than accurate patients (*P* = 0.002). For both types of symptom sensitivity, stoical patients showed significantly less anxiety and depression and reported significantly higher quality of life than both sensitive and accurate patients (*P* < 0.05 for all). There were no significant differences in anxiety and depression between the sensitive group and the accurate group (*P* > 0.05 for all). There was a significant difference between the groups in the self‐reported presence of functional conditions, with the sensitive group reporting a significantly higher incidence of both fibromyalgia and irritable bowel syndrome than the stoical group (*P* < 0.017 for all). Oral‐sensitive patients reported a significantly higher incidence of irritable bowel syndrome than accurate patients (*P* = 0.003), but otherwise there were no further significant differences between the sensitive and accurate groups. There were no significant differences between the groups in the proportion of those with any Diagnostic and Statistical Manual of Mental Disorders–defined or International Classification of Diseases–defined mental illness (*P* > 0.05 for all).

**Table 2:** Mean comorbidities, no. medications, and scores for depression, anxiety, and quality of life by dryness sensitivity classification. Proportions of patients in the different groups with specific comorbidities and receiving particular treatments^a^

	Ocular sensitivity					Oral sensitivity				
	Stoical (n = 351)	Accurate (n = 130)	Sensitive (n = 200)	Comparison	*P*	Stoical (n = 444)	Accurate (n = 179)	Sensitive (n = 58)	Comparison	*P*
No. comorbidities	3.4 ± 2.3	3.9 ± 2.5	3.8 ± 2.6	F_2,678_ = 2.90	0.056	3.4 ± 2.3	3.8 ± 2.6	3.9 ± 2.7	F_2,678_ = 9.5	0.078
No. medications	5.5 ± 4.1	5.7 ± 3.7	6.3 ± 4.2	F_2,678_ = 2.52	0.081	5.4 ± 3.9^b^	6.6 ± 4.5^c^	5.8 ± 3.9^d^	F_2,678_ = 4.97^e^	0.007^e^
Anxiety (HADS)	7.3 ± 4.5^b^	8.4 ± 4.8^c^	8.9 ± 4.4^c^	F_2,661_ = 8.75^e^	< 0.001^e^	7.3 ± 4.4^b^	9.0 ± 4.8^c^	10.2 ± 4.0^c^	F_2,661_ = 16.45^e^	< 0.001^e^
Depression (HADS)	5.3 ± 3.7^b^	6.4 ± 4.3^c^	6.9 ± 4.3^c^	F_2,661_ = 11.23^e^	< 0.001^e^	5.2 ± 3.6^b^	7.2 ± 4.4^c^	8.1 ± 4.3^c^	F_2,661_ = 24.00^e^	< 0.001^e^
Quality of life (VAS)	63.8 ± 20.6^b^	56.6 ± 22.5^c^	56.3 ± 21.2^c^	F_2,659_ = 10.09^e^	< 0.001^e^	63.4 ± 20.2^b^	54.3 ± 22.5^c^	53.9 ± 22.7^c^	F_2,659_ = 14.14^e^	< 0.001^e^
Specific comorbidities										
Fibromyalgia	6.3^c^	8.5^d^	14.0^b^	Χ^2^(2) = 9.39^e^	0.009^e^	7.0^c^	11.2^d^	17.2^b^	Χ^2^(2) = 8.08^e^	0.018^e^
IBS	5.4^c^	7.7^d^	12.0^b^	Χ^2^(2) = 7.70^e^	0.021^e^	7.7^c^	5.0^c^	17.2^b^	Χ^2^(2) = 9.13^e^	0.010^e^
Mental illness	3.1	6.2	4.0	Χ^2^(2) = 2.27	0.321	2.7	6.7	5.2	Χ^2^(2) = 5.61	0.061
Treatments, %										
Xerogenic medication use	37.3^b^	46.2^d^	50.0^c^	Χ^2^(2) = 9.14^e^	0.010^e^	39.2	48.6	51.7	Χ^2^(2) = 6.72^e^	0.035^e^
Symptomatic treatment for dryness	97.7	96.9	97.5	Χ^2^(2) = 0.25	0.883	97.1	97.8	100.0	Χ^2^(2) = 1.875	0.392
Pilocarpine	7.1	9.2	7.5	Χ^2^(2) = 0.61	0.739	7.2	10.1	3.4	Χ^2^(2) = 3.04	0.218
Saliva substitute	–	–	–	–		40.1^b^	56.4^c^	43.1^d^	Χ^2^(2) = 13.83^e^	0.001^e^
Lachrymal substitute	82.6^c^	83.1^c^	71.5^b^	Χ^2^(2) = 10.95^e^	0.004^e^	–	–	–	–	
Punctal plugging or cauterization	18.8^b^	33.8^c^	22.0^d^	Χ^2^(2) = 12.33^e^	0.002^e^	–	–	–	–	

a Values are the mean ± SD unless otherwise indicated. HADS = Hospital Anxiety and Depression Scale; VAS = visual analog scale; IBS = irritable bowel syndrome.

b Significantly different from groups ‡ and § in post hoc tests (*P* < 0.05 for continuous measures, *P* < 0.017 for categorical measures).

c Significantly different from groups † and § in post hoc tests (*P* < 0.05 for continuous measures, *P* < 0.017 for categorical measures).

d Significantly different from groups † and ‡ in post hoc tests (*P* < 0.05 for continuous measures, *P* < 0.017 for categorical measures).

e Statistically significant.

Spearman's bivariate correlations were used to explore other factors associated with symptom sensitivity, and the results for both ocular and oral dryness sensitivity are shown in Table 3. Ocular sensitivity was weakly positively correlated with number of medications and weakly negatively correlated with age, suggesting that patients taking fewer medications and older patients were more stoical. Oral sensitivity was weakly positively correlated with number of comorbidities and number of medications, suggesting that those with fewer comorbidities and taking fewer medications were more stoical. Both types of sensitivity were moderately strongly positively correlated with patient‐rated symptoms, including fatigue, mental fatigue, pain, anxiety, and depression. The direction of the correlations indicated that those with higher levels of these symptoms were more sensitive. Both types of sensitivity were negatively correlated with quality of life, indicating that those with poorer quality of life had higher sensitivity scores.

**Table 3:** Spearman's (r_s_) bivariate correlations between ocular and oral sensitivity^a^

	Ocular sensitivity	Oral sensitivity
Demographic and illness factors		
Age	−0.11^b^	−0.02
Disease duration	0.02	0.07
Symptom duration	−0.00	0.03
No. comorbidities	0.07	0.11^c^
No. medications	0.10^c^	0.12^c^
Patient‐rated measures		
Fatigue	0.38^b^	0.39^b^
Mental fatigue	0.33^b^	0.30^b^
Pain	0.33^b^	0.29^b^
Quality of life, time tradeoff	−0.25^b^	−0.24^b^
Quality of life, VAS	−0.21^b^	−0.28^b^
HADS anxiety	0.20^b^	0.26^b^
HADS depression	0.22^b^	0.30^b^
Clinician‐rated measures		
ESSDAI	0.02	0.03
ESSDDI	−0.07	0.05
Oral sensitivity	0.35^b^	–

a VAS = visual analog scale; HADS = Hospital Anxiety and Depression Scale; ESSDAI = European League Against Rheumatism (EULAR) Sjögren's Syndrome Disease Activity Index; ESSDDI = EULAR Sjögren's Syndrome Disease Damage Index.

b *P* ≤ 0.001.

c *P* from 0.001 to ≤ 0.01.

### Relationship between treatment and symptom sensitivity

Patients in the different sensitivity classes receiving treatment were examined in order to explore relationships between symptom sensitivity and treatment received (Table 2). Ocular‐stoical patients were significantly less likely to be receiving a medication known to cause dryness than sensitive patients (*P* = 0.004), and while there was an overall group difference for oral sensitivity, post hoc tests did not show any significant differences between the groups. There were no significant differences between the groups in the use of at least 1 symptomatic treatment for dryness or pilocarpine (*P* > 0.218 for all). However, oral stoics were significantly less likely to be using a saliva substitute (40.1%) than accurate patients (56.4%; *P* < 0.001), and there was no significant difference between stoical and sensitive patients (43.1%; *P* = 0.660). Similarly for ocular sensitivity, ocular stoics were significantly less likely than accurate patients to have received the more invasive treatments of punctal plugging or cauterization (18.8% versus 33.8%; *P* = 0.012), and there were no differences in ocular‐sensitive patients (22.0%; *P* = 0.367). Ocular‐sensitive patients were significantly less likely to be using a lachrymal substitute than both the accurate and stoical patients (71.5% versus 83.1% and 82.6%, respectively; *P* = 0.016 and *P* = 0.002, respectively).

### Regression analysis

#### Ocular dryness

The final model contained 6 predictor variables and was statistically significant (F_6,628_ = 21.8, *P* < 0.001), explaining 16.5% of the variance in sensitivity (Table 4). The statistically significant predictors in the final model were age, ESSDDI score, pain, fatigue, and mental fatigue. Pain explained the largest additional variance (10%) of all the predictors, and fatigue was the next highest (2.3%). Age and ESSDDI score were both negatively related to sensitivity, indicating that older patients and those with greater disease damage tended to be less sensitive.

#### Oral dryness

The final model contained 6 predictor variables and was statistically significant (F_6,628_ = 24.1, *P* < 0.001) explaining 17.9% of the variance in sensitivity (Table 4). The statistically significant predictors in the final model were use of a saliva substitute, fatigue, and depression, all of which were positively associated with greater sensitivity. Level of fatigue explained the largest additional variance (11.7%), indicating that those with higher fatigue tended to be more sensitive.

**Table 4:** Stepwise hierarchical regression summary for ocular dryness sensitivity and oral dryness sensitivity^a^

Model	Adj. R^2^ (%)	ΔR^2^ (%)	Standardized β	Test‐statistic	*P*
Ocular dryness sensitivity					
Overall model	16.5	–	–	F_6,628_ = 21.8	< 0.001^b^
Age		1.3	−0.09	t_634_ = −2.39	0.017^c^
No. medications		1.9	0.01	t_634_ = 0.25	0.802
ESSDAI		0.5	−0.08	t_634_ = −2.23	0.026^c^
Pain		10.0	0.19	t_634_ = 3.89	< 0.001^b^
Fatigue		2.3	0.16	t_634_ = 2.95	0.003^b^
Mental fatigue		0.5	0.11	t_634_ = 2.14	0.033^c^
Oral dryness sensitivity					
Overall model	17.9	–	–	F_6,628_ = 24.1	< 0.001^b^
Use of saliva substitute	–	2.9	0.15	t_634_ = 3.99	< 0.001^b^
No. comorbidities	–	1.3	0.02	t_634_ = 0.43	0.667
Age	–	0.6	−0.01	t_634_ = −0.21	0.831
Xerogenic medications	–	0.5	0.01	t_634_ = 0.24	0.812
Fatigue	–	11.7	0.30	t_634_ = 6.88	< 0.001^b^
Depression	–	0.9	0.13	t_634_ = 2.91	0.004^b^

a Adj. = adjusted; ESSDAI = European League Against Rheumatism Sjögren's Sydrome Disease Activity Index.

b *P* ≤ 0.001.

c *P* > 0.001 and ≤0.05.

## Discussion

As in other studies, subjective dryness symptoms and objective test results were only weakly correlated in patients with primary SS. A novel method for quantifying the discrepancy between subjective and objective symptoms was devised, as an ordinal scale of symptom sensitivity, ranging from stoical (self‐reported dryness at a relatively low level compared to objective findings) to accurate to sensitive (self‐reported dryness at a relatively high level compared to objective findings). The majority of patients had a relatively stoical presentation. A significant moderate association was observed between ocular and oral dryness sensitivity, indicating that those who tended to be sensitive to ocular dryness also tended to be sensitive to oral dryness. Comparisons of sensitive, stoical, and accurate patients showed that stoical patients had lower depression and anxiety scores than the other groups, but they were also less likely to have received some treatments than accurate patients (saliva substitute and punctal plugging or cauterization). Sensitive patients were not more likely to receive higher levels of intervention than accurate or stoical patients, but a greater proportion of them reported functional conditions (fibromyalgia and irritable bowel syndrome). In regression analyses, symptom sensitivity was predicted by a variety of factors, but pain (ocular sensitivity) and fatigue (oral sensitivity) explained the most variance.

As found in other studies, the relationship between subjective and objective measures was weaker for ocular dryness than for oral dryness 35, 36, 37. Ocular dryness sensitivity was predicted by greater pain and fatigue, whereas age and disease damage were significant negative predictors, suggesting that older patients and those with more severe disease are relatively more stoical. Adatia et al 7 suggested that symptom perception may be diminished by reduced corneal sensation due to more severe illness, which may explain the negative relationship with disease damage. This may be part of the explanation for why subjective and objective ocular dryness measures correlate relatively more weakly; a straightforward, linear relationship between severity of disease and severity of subjective symptoms would not be expected. Additionally, there were fewer patients whose objective test results fell in the severe range for tear production (46%) compared to saliva production (77%), and 16% had objectively normal tear production (compared to only 4% for saliva production), leaving more scope to identify ocular patients as sensitive. The symptom of dry eye is less well‐defined than dry mouth and may be used to refer to a myriad of ocular sensations, including burning pain, grittiness, and tired or heavy eyes. This introduces heterogeneity in patients, in terms of what they mean when they report having dry eyes, and not all of the experienced sensations can be expected to relate to tear production. As “dry eye” often refers to painful sensations in the eye, this may explain why self‐reported pain was the greatest predictor of ocular sensitivity (but was not a significant predictor of oral sensitivity).

In contrast, sensitivity to oral dryness symptoms was most strongly associated with global fatigue, followed by use of a saliva substitute and number of comorbidities. It was not related to disease severity or pain. This suggests that different processes are related to symptom sensitivity to different symptoms. The overall proportion of variance explained in both regression models (i.e., 16.5–17.9%) indicates that there are explanatory factors that were not included in the present study, which should be explored in future studies. Biologic factors relating to the composition of the tears or saliva may be relevant. Xerostomia can be affected by saliva composition 38, and multiple factors, such as lachrymal secretion, corneal damage, tear film stability, and the chemical properties of tears all jointly affect the perception of ocular dryness 39. Relatively sensitive individuals could be targeted in future research to identify biologic markers in tears or saliva that may affect perceived dryness.

Psychologic models of symptom perception propose a large number of factors that may impact on whether someone notices a symptom 26 and could be of relevance to measure in PSS. Trait characteristics such as neuroticism, alexithymia (the ease with which one identifies emotions), and distress tolerance may play a role 40, 41. It has been shown that catastrophization (a manner of thinking that exaggerates worries and amplifies negative consequences) 42 is highly predictive of pain severity in patients with primary SS 43. Similarly, greater body‐focused attention may contribute to symptom‐noticing 44, and somatosensory amplification (a heightened responsiveness to sensory stimulation) has been shown to contribute to the symptoms of many rheumatic conditions 41. Geisser et al 45 found that amplification in chronic fatigue syndrome and fibromyalgia was related to higher clinical pain and larger numbers of comorbid somatic symptoms and, consistent with this finding, we identified a greater proportion of patients with fibromyalgia in the sensitive groups. Anxiety and depression have also been shown to be significantly related to greater sensitivity. In a population‐based study by Anttila et al 22, participants with subjective dry mouth had depressive symptoms in significantly higher frequencies. Similarly, Kim et al 24 concluded that depression was associated with dry eye symptoms in participants with normal Schirmer test results. Additionally, social, contextual, cultural, and interpersonal factors likely also contribute to how and whether patients openly discuss their symptoms with their doctor, making it difficult to determine, particularly for stoical patients, whether they do not experience distressing symptoms or they simply do not report them.

The strengths of this study include the large sample of primary SS patients and the novel method for defining sensitivity, which allowed potential associated variables to be investigated. However, there are a number of limitations. First, self‐report methods are prone to response bias, such as demand characteristics bias, social desirability bias, and recall bias 46. Furthermore, when completing the self‐report measure, participants judged the severity of their sicca symptoms against their own standards; however, the severity of objective measures is judged against standards formulated from the results of many individuals, so a degree of discrepancy could be expected. Second, only 1 objective measure of ocular and oral dryness was used. USF is the test of choice for assessing salivary secretion 20; however, variations of methods exist for measuring ocular dryness. While the Schirmer I test is a valid assessment, issues regarding reproducibility and sensitivity have been reported 47, and its use without anesthesia (as in the present study) includes both basal and reflex lachrymal secretion 48, which may exaggerate the severity of objective ocular dryness in those with progressive corneal desensitization.

Categories of objective symptoms were derived specifically for this study, in order to calculate the sensitivity score. While the expert opinion of a consulting rheumatologist was used in developing the categories and exploratory work using different cutoff scores or different ways of categorizing patients showed the same pattern of relationships, the categories used here need further empirical support and replication in future studies to determine whether or not they can be operationalized and used clinically.

Going forward, we advocate the use of a measure of objective‐subjective symptom discordance, such as that outlined here, to facilitate illness stratification to allow for further research into the reasons behind this. Of particular interest are the groups with the greatest discordance, i.e., patients reporting severe subjective symptoms despite being at the milder end of the objective symptom distribution, and those reporting mild subjective symptoms despite being at the severe end of the objective distribution. An exploration of potential physical and psychologic explanations for this phenomenon is warranted. For example, is there a difference in pathophysiology that might contribute to the increased experience of mildly abnormal objective symptoms, such as tear and saliva composition, changes in corneal sensitivity, or genetic differences in, for example, pain sensitivity? Conversely, there is detailed literature on the psychologic aspects of interoception and pain perception. Applying some of the methodologies from this literature to develop our understanding of the individual differences in the felt experience of physical symptoms would allow us to explore alternative treatment options, such as cognitive–behavioral therapy or mindfulness for those with lower symptom tolerance/higher subjective symptom distress 49, 50, 51.

In conclusion, the study developed a novel method for determining symptom sensitivity. Discrepancies between objective measures and subjective symptoms were most strongly related to pain and fatigue; however, multiple interrelated psychologic, pathophysiologic, and environmental factors are likely involved. Limitations associated with accurately measuring dry eyes/mouth both subjectively and objectively may also contribute to the observed discrepancies. Stratifying patients by symptom sensitivity for further research will improve our understanding of factors that have an impact on distress caused by symptoms and could open the door to nonmedication‐based treatments for a subgroup of patients with the most sensitivity to symptoms.

## Author Contributions

All authors were involved in drafting the article or revising it critically for important intellectual content, and all authors approved the final version to be submitted for publication. Dr. Robinson had full access to all of the data in the study and takes responsibility for the integrity of the data and the accuracy of the data analysis.

### Study conception and design

Bezzina, Gallagher, Ng, Robinson.

### Acquisition of data

Bezzina, Gallagher, Mitchell, Bowman, Griffiths, Hindmarsh, Hargreaves, Price, Pease, Emery, Lanyon, Bombardieri, Sutcliffe, Pitzalis, Hunter, Gupta, McLaren, Cooper, Regan, Giles, Isenberg, Saravanan, Coady, Dasgupta, McHugh, Young‐Min, Moots, Gendi, Akil, MacKay, Ng, Robinson.

### Analysis and interpretation of data

Bezzina, Gallagher, Mitchell, Bowman, Griffiths, Hindmarsh, Hargreaves, Price, Pease, Emery, Lanyon, Bombardieri, Sutcliffe, Pitzalis, Hunter, Gupta, McLaren, Cooper, Regan, Giles, Isenberg, Saravanan, Coady, Dasgupta, McHugh, Young‐Min, Moots, Gendi, Akil, MacKay, Ng, Robinson.

## Members of the UK Primary Sjögren's Syndrome Registry

Simon J. Bowman, Bhaskar Dasgupta, and W. Fai Ng are UK Primary Sjögren's Syndrome Registry investigators. The other UK Primary Sjögren's Syndrome Registry members (as of January 1, 2013) are as follows: Frances Hall (Addenbrooke's Hospital, Cambridge); Elalaine C. Bacabac, Robert Moots (Aintree University Hospitals); Kuntal Chakravarty, Shamin Lamabadusuriya (Barking, Havering, and Redbridge NHS Trust); Michele Bombardieri, Constantino Pitzalis, Nurhan Sutcliffe (Bart and the London NHS Trust); Nagui Gendi, Rashidat Adeniba (Basildon Hospital); John Hamburger, Andrea Richards (Birmingham Dental Hospital); Saaeha Rauz (Birmingham & Midland Eye Centre); Sue Brailsford (University Hospitals Birmingham); Joanne Logan, Diarmuid Mulherin (Cannock Chase Hospital); Jacqueline Andrews, Paul Emery, Alison McManus, Colin Pease (Chapel Allerton Hospital, Leeds); Alison Booth, Marian Regan (Royal Derby Hospital); Theodoros Dimitroulas, Lucy Kadiki, Daljit Kaur, George Kitas (Dudley Group of Hospitals NHS Foundation Trust); Mark Lloyd, Lisa Moore (Frimley Park Hospital); Esther Gordon, Cathy Lawson (Harrogate District Foundation Trust Hospital); Monica Gupta, John Hunter, Lesley Stirton (Gartnavel General Hospital, Glasgow); Gill Ortiz, Elizabeth Price (Great Western Hospital); Gavin Clunie, Ginny Rose, Sue Cuckow (Ipswich Hospital NHS Trust); Susan Knight, Deborah Symmons, Beverley Jones (Macclesfield District General Hospital & Arthritis Research UK Epidemiology Unit, Manchester); Shereen Al‐Ali, Andrew Carr, Katherine Collins, Ian Corbett, Christine Downie, Suzanne Edgar, Marco Carrozzo, Francisco Figuereido, Heather Foggo, Ben Hargreaves, Victoria Hindmarsh, Claire Humphreys, Katherine James, Dennis Lendrem, James Locke, Iain Macleod, Philip Mawson, Sheryl Mitchell, Philip Stocks, Jessica Tarn (Newcastle upon Tyne Hospitals NHS Foundation Trust and Newcastle University); Adrian Jones, Peter Lanyon, Alice Muir (Nottingham University Hospital); Paula White, Steven Young‐Min (Portsmouth Hospitals NHS Trust); Susan Pugmire, Saravanan Vadivelu (Queen's Elizabeth Hospital, Gateshead); Annie Cooper, Marianne Watkins (Royal Hampshire County Hospital); Anne Field, Stephen Kaye, Devesh Mewar, Patricia Medcalf, Pamela Tomlinson, Debbie Whiteside (Royal Liverpool University Hospital); Neil McHugh, John Pauling, Julie James, Nike Olaitan (Royal National Hospital for Rheumatic Diseases); Mohammed Akil, Jayne McDermott, Olivia Godia (Royal Sheffield Hospital); David Coady, Elizabeth Kidd, Lynne Palmer (Sunderland Royal Hospital); Bhaskar Dasgupta, Victoria Katsande, Pamela Long (Southend University Hospital); Charles Li (Royal Surrey Hospital); Usha Chandra, Kirsten MacKay (Torbay Hospital); Stefano Fedele, Ada Ferenkeh‐Koroma, Ian Giles, David Isenberg, Helena Maconnell, Stephen Porter (University College Hospital and Eastman Dental Institute); Paul Allcoat, John McLaren (Whyteman's Brae Hospital, Kirkcaldy).
